# Experimental Inoculation of BFDV-Positive Budgerigars (*Melopsittacus undulatus*) with Two *Mycobacterium avium* subsp. *avium* Isolates

**DOI:** 10.1155/2014/418563

**Published:** 2014-03-13

**Authors:** Aleksandra Ledwoń, Rafał Sapierzyński, Ewa Augustynowicz-Kopeć, Piotr Szeleszczuk, Marcin Kozak

**Affiliations:** ^1^Department of Pathology and Veterinary Diagnostics, Warsaw University of Life Sciences, Nowoursynowska 159c Street, 02-776 Warsaw, Poland; ^2^Department of Microbiology, Institute of Tuberculosis and Pulmonary Diseases, Płocka 26 Street, 01-138 Warsaw, Poland; ^3^University of Information Technology and Management in Rzeszow, H. Sucharskiego 2 Street, 35-225 Rzeszow, Poland

## Abstract

Beak and feather disease virus- (BFDV-) positive (naturally infected) but clinically healthy budgerigars (*Melopsittacus undulatus*) were inoculated with two isolates of *Mycobacterium avium* subsp. *avium* isolated from naturally infected golden pheasant (*Chrysolophus pictus*) and peafowl (*Pavo cristatus*). During a period of more than two months after inoculation, samples of cloacal and crop swabs, faeces, and blood were obtained for BFDV and *Mycobacterium avium* testing with PCR. Birds were euthanized nine weeks after inoculation. All infected budgerigars developed signs typical of mycobacteriosis, but more advanced clinical and pathological changes were visible in the group infected with the pheasant isolate. Only a few cloacal and crop swab samples were positive for *Mycobacterium avium* subsp. *avium* despite advanced pathological changes in the internal organs. In the groups infected with mycobacterium isolates the frequency of BFDV-positive samples was higher than in the control group. In the infected groups the frequency of BFDV was substantially higher in the cloacal swabs of birds inoculated with the pheasant isolate than in the peafowl-isolate-infected group.

## 1. Introduction

Mycobacterioses in pet birds occur with constant prevalence which can be even more than 1% of total necropsy submissions [1]. They are mainly caused by* Mycobacterium avium* subsp.* avium* [2, 3] and* Mycobacterium genavense* [4, 5].

Mycobacterioses are often correlated with immunosuppression, which can be caused by viral agents [6]. In psittacine birds the most important immunosuppressive viral disease is psittacine beak and feather disease—PBFD—which is caused by the beak and feather disease circovirus (BFDV) [7–10]. Circovirus will selectively attack the thymus and bursa of Fabricius preventing lymphocyte production and severely impairing the bird's immune system. The younger the bird is infected the more severe the immunosuppression is. Normally birds develop the antibody diversity in the bursa Fabricius during first 3-6 weeks of their lives provided no infection had taken place before this period, otherwise an adequate immune system will be never established. These birds with a suppressed immune system due to PBFD will commonly suffer from a range of secondary infections [11].

A psittacine circoviral infection shows a relatively high degree of spread throughout parrot colonies. The virus was also detected in birds free of clinical signs [7–9].

We used BFDV-positive budgerigars for the experimental infection with* Mycobacterium avium* subsp.* avium* to check relations as to the presence of these viral and mycobacterial pathogens in the crop and cloacal swabs as well as blood and faeces.

## 2. Material and Methods

### 2.1. Course of an Experiment

The authors obtained a positive opinion from the Local Ethics Commission (nr 39/2008), prior to using budgerigars in the experiment. A total of 18 of about-six-month-old budgerigars were used. Birds with no signs of illness were randomly assigned to experimental groups, each consisting of six individuals. The budgerigars were fed a commercial seed mix, Prestige Premium (Versele Laga, Belgium), supplemented with vitamin mixture Vinka (Beaphar, The Netherlands) and cuttlefish bone (Vadigran, Belgium). Parakeets were fed* ad libitum*.

Budgerigars were inoculated with* M. avium* subsp.* avium. *Two experimental groups were created. GroupPh was inoculated withmycobacteria isolated from a necropsied golden pheasant (*Chrysolophus pictus*) with advanced mycobacteriosis. The second group P was infected with* Mycobacterium avium* subsp.* avium* isolated from Indian peafowl (*Pavo cristatus*) with respiratory mycobacteriosis. Both* Mycobacterium avium* subsp.* avium* isolates were cultured on BBL Lowenstein-Jensen Medium + PACT (Becton Dickinson, USA) for 4 weeks in 37°C. The inoculum was administered into the pectoral muscles at a dose of about 5 × 10^5^ colony-forming units/kg body weight [12]. Another six birds comprised the negative control group.

During the experimental period, body weight control and cloacal and crop swabs were obtained weekly and blood samples from the right jugular vein were obtained every second week. Swabs and blood samples were tested by QPCR. Before the start of experiment feather samples were tested for BFDV by PCR.

The birds were submitted to euthanasia 10 weeks after inoculation. Euthanasia was performed using pentobarbital sodium (Morbital Biowet Pulawy, Poland) intravenously. During necropsy, samples of the proventriculus, gizzard, intestine, pancreas, heart, lung, pectoral muscle, brain, kidney, and gonads were collected for histopathological examinations. Tissue samples were fixed in 10% buffered formalin. The fixed tissue samples were stained with haematoxylin and eosin stain or according to the Ziehl-Neelsen method.

### 2.2. DNA Extraction

DNA was extracted from crop and cloacal swab samples using the Swab 100 DNA extraction kit (A&A Biotechnology, Poland) according to the manufacturer's instructions with the exception of time of incubation, which was prolonged to two hours.DNA extraction from the feathers and blood was performed with 5% Chelex (Bio-Rad Laboratories, Canada) [13].

### 2.3. Primers

BFDV PCR was performed according to Katoh et al. [14]. A selection of specific* Mycobacterium avium* subsp.* avium* primers was supported by Bacon Designer software 7 (PREMIER Biosoft International, Canada) on* Mycobacterium avium* subsp.* avium* ATCC 25291 NZ_ACFI01000238 [15]. The chosen forward primer MAA-s (ACACCGTCAGCATCAAGG, Tm°C: 53.7) was located at nucleotides 489 to 506* Mycobacterium avium* subsp.* avium*, and MAA-as (GAAGTTAGCGGAAATTCAAGC, Tm°C: 53.2) corresponded to nucleotides 588 to 608. BFDV in feather samples was detected with PCR according to Ypelaar et al. [16].

### 2.4. Positive Controls

The positive control for BFDV was DNA isolated from the feathers of parrots with clinical signs of PBFD. This test was considered only as qualitative.* M. avium* subsp.* avium *DNA was obtained from a pure culture of* Mycobacterium avium* subsp.* avium* using the Sherlock AX kit (A&A Biotechnology, Poland) according to the manufacturer's instructions. The quantity of DNA was measured with NanoDrop (NanoDrop Technologies, USA) to ng/*μ*L and recounted to the number of DNA copies with Stratagene Mx3005, MxPro QPCR Software, 2007.

### 2.5. Real-Time PCR Assay

Real-time PCR amplification was carried out in a total volume of 25 *μ*L using Brilliant SYBR Green QPCR Master Mix (Stratagene, Canada) containing 0.5 *μ*L of each primer and 3 *μ*L of the DNA template. The PCR was performed in the Stratagene Mx3005PTM cycler (Stratagene, Canada). BFDV reactions were performed according to published data [14].* Mycobacterium avium* subsp.* avium* QPCR was performedwith the following protocol: initial denaturation for 10 min at 95°C followed by 40 cycles consisting of denaturation at 95°C for 30 s, annealing at 55°C for 60 s, and elongation at 72°C for 60 s. Fluorescence data were collected during the elongation step. After termination of the reaction by a final extension step at 72°C for 5 min, a DNA-melting curve was generated to verify the correct product by its specific melting temperature. Melting-curve analysis was performed by heating at 95°C for 1 min, followed by cooling to 55°C for 30 s and subsequent heating to 95°C for 30 s. For each real-time PCR reaction, software associated with the Stratagene Mx3005PTM system determined a threshold of the cycle number (C_*t*_). The specific melting temperature value of the real-time product was about 76.8°C.

To determine the sensitivity of the real-time PCR assay, 11-fold serial dilutions of positive control DNA ranging from 2.86 × 10^10^ to 2.86 × 10^0^ of DNA copies were tested. C_*t*_ value range was 19.7 (2.86 × 10^10^) to 40 (2.86 × 10^1^).

### 2.6. Statistics

General linear models with a binomial link function [17] were used to compare the three groups in terms of BFDV and* M. avium*. For the analysis, the repeated measures character of the data was ignored, and what was of interest to us was how many samples were positive out of all the samples for a particular bird. Multiple comparisons for generalized linear models [18] were used when the general hypothesis of the lack of a difference among the groups was rejected.

## 3. Results

### 3.1. Clinical Observations

Despite the partial lack of remiges in a few birds there was an absence of the clinical signs of PBFD disease. The first symptoms of mycobacteriosis appeared in group Ph (infected with the pheasant isolate of* M. avium*) 3 weeks after infection as moderate polyuria. Five weeks after infection a yellow discolouration of the ureates and in week 8 excessive polyuria with green ureates was observed. In week 9 diarrhoea appeared in one budgerigar and one week later this bird died. In group Pa the first clinical signs of yellow urine discolouration appeared in one budgerigar in week 9; polyuria and biliverdinuria in all Pa parakeets were present in week 10. Body mass in the three groups was compared by means of linear mixed-effects models [19] in which observations of body mass were nested within particular birds (Table 1).

### 3.2. Pathology

**Figure 1:**
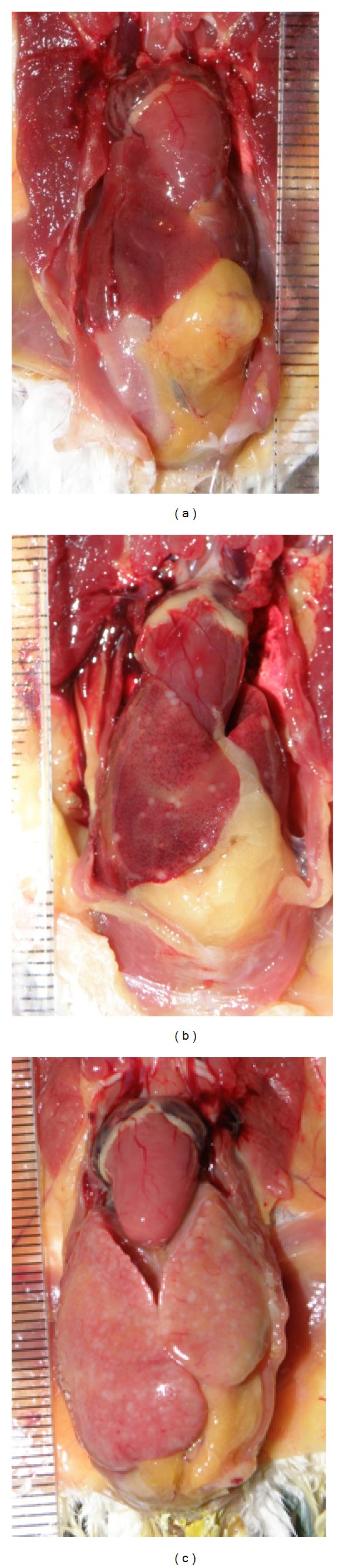
Liver necropsy (groups: (a) control; (b) infected with* M. avium* subsp.* avium: *peafowl isolate; (c) infected with* M. avium* subsp.* avium: * pheasant isolate).

Typical advanced changes were observed in the liver (Figure 1) and spleen just as in the place of inoculation in all of the necropsied birds from the Ph group. Miliary abscesses were observed in the liver and marked hepatomegaly was observed in five birds.In birds infected with the peafowl originating isolate (Pagroup) the lesions were less prominent but typical of avian mycobacteriosis.

Prevalence and severity of typical of mycobacterioses histopathological changes are shown in Table 2. Other abnormalities also found in the control group were splenic white pulp proliferation and microvesicular steatosis. Splenic white pulp proliferation was observed in 4/6 of birds in the control group, 2/6 in the Pa group, and 2/6 in the Ph group; microvesicular steatosis was observed in 3/6 of the control group, 3/6 of the Pagroup, and 1/6 of the Phgroup.

All of the infected budgerigars developed changes typical of mycobacteriosis, but more advanced pathological changes were visible in the group infected with the pheasant isolate. Five out of 18 samples were BFDV negative, during the experiment, despite the fact that all of the birds were in the same room and the spread of the virus undoubtedly took place. One parakeet with negative feather samples was only once a blood-positive bird, in the Ph group; another bird from the control group was blood negative during the course of the experiment (Table 3).

For the two groups, K and Ph, M*. avium* was not detected at all, and for Pa it was detected in only a few samples (Table 4).

## 4. Discussion

In our previous experiment, published in 2008, budgerigars were infected with* Mycobacterium avium *subsp*. avium*, which caused no clinical or pathological changes typical of mycobacteriosis [12]. In the present study when we used the same amount of bacteria in the same environmental conditions, advanced mycobacteriosis was endangered. The most important factor was probably bacteria pathogenicity. Isolates used in the current study were isolated from lethal cases of spontaneous mycobacteriosis in gallinaceous birds and were used shortly after isolation from the tissues. Strain and isolate used in the previous study originated from the collection of the National Tuberculosis and Lung Diseases Research Institute in Warsaw or were isolated from the faeces of healthy birds. Another important reason was the subclinical viral infections in the budgerigars which affected their immunological system. However, the histopathology was not typical of circoviral infections. Microscopy of the spleen in some of the control as well as infected birds revealed the proliferation of white pulp, which can be consistent with the presence of a chronic inflammation. By contrast, circoviruses commonly cause lymphocyte depletion and spleen atrophy [20].

**Figure 2:**
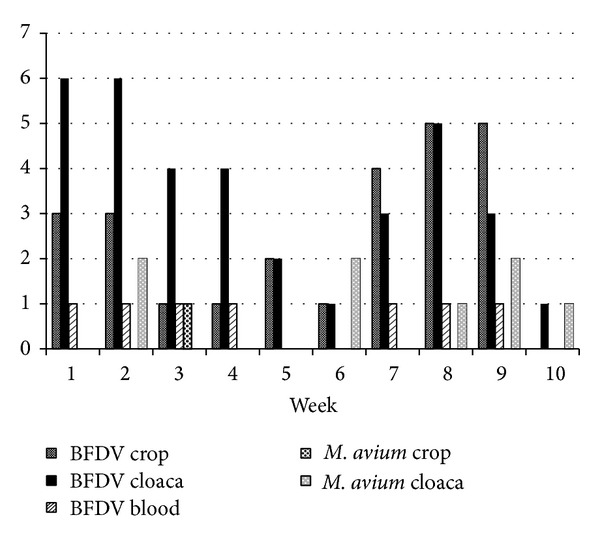
Frequency of mycobacteria and BFDV-positive samples in budgerigars infected with the peafowl isolate of* M. avium* subsp.* avium*.

**Figure 3:**
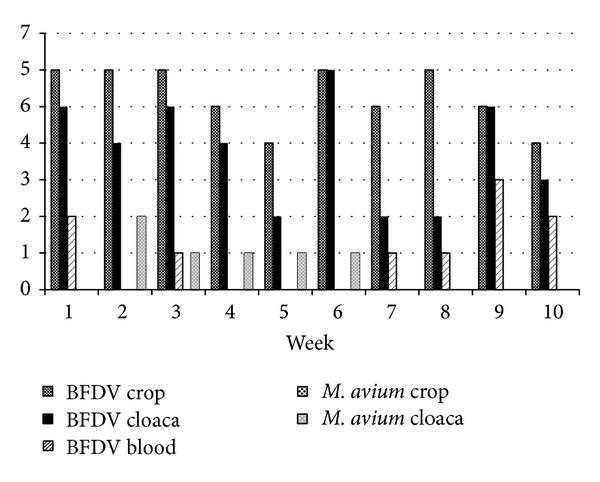
Frequency of mycobacteria and BFDV-positive samples in budgerigars infected with the pheasant isolate of* M. avium* subsp.* avium*.

**Figure 4:**
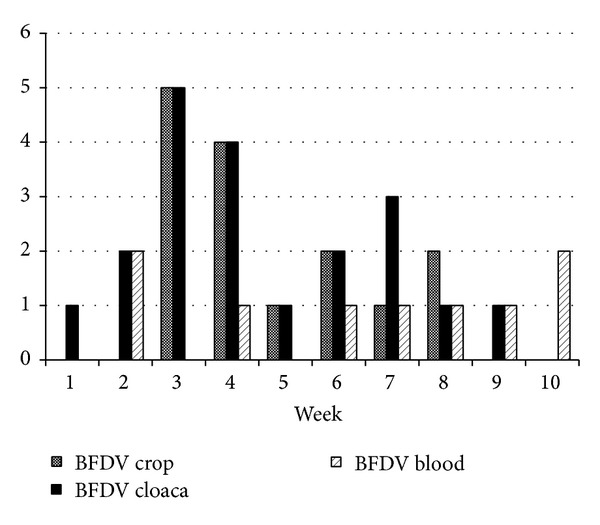
Frequency of mycobacteria and BFDV-positive samples in the control group of budgerigars.

Yet circovirus shedding was correlated with mycobacterial infection (Figures 2, 3, and 4). Budgerigars inoculated with pheasant isolate of* Mycobacterium avium* subsp.* avium *were more frequently BFDV positive (Table 2) than with peafowl isolate (Table 3) and control group, respectively. Therefore an important finding is that a chronic bacterial infection depending on it severity can cause excess of viral particles shedding. The research can also be used to evaluate QPCR for diagnostics of mycobacterioses in live birds. In human patients tuberculosis sputum samples are the most commonly examined [21], whereas in our research cloacal and crop swabs were tested. Only a few samples were positive despite the advanced pathological changes in the internal organs. Our previous study involving other species of mycobacteria cultures of faeces proved also unsatisfactory in terms of the anticipated number of positive samples [12]. Thus, cloacal and crop swabs do not constitute valuable material for diagnostics of* Mycobacterium avium* subsp.* avium* in budgerigars.

## Figures and Tables

**Table 1:** Body mass analysis.

Group	Values mean
Pa	44.0^a1^
K	40.5^a^
Ph	40.3^a^

^1^The same letters in a column mean that birds within the three groups studied did not significantly differ in body mass (*P* = 0.606).

**Table 2:** Prevalence of typical mycobacteriosis histopathological changes: g, granulomas; i, infiltration of granulocytes; if, inflammation; n, necrosis.

Organ	Group Ph	Group P	Control
Liver	6 (g)	4 (g) 1 (if)	**—**
Spleen	1 (g) 3 (i)	1 (i)	—
Proventriculus	1 (i)	—	—
Gizzard	—	—	—
Intestine	—	1 (if)	—
Pancreas	—	—	—
Heart	2 (i)	—	—
Lung	1 (if, n)	—	—
Pectoral muscle	5 (g) 1 (i)	5 (g)	—
Brain	—	—	—
Kidney	2 (i)	—	—
Gonads	—	—	—

**Table 3:** Occurrence of BFDV-positive samples.

Group	Crop swab	Cloacal swab	Blood
P	0.491^b1^	0.660^b^	0.333
Ph	0.900^c^	0.600^b^	0.267^a^
K	0.267^a^	0.333^a^	0.217^a^

^1^The different letters in the column represent a different mean share of the positive samples for birds within the two corresponding groups.

**Table 4:** Occurrence of *M. avium* positive samples.

Group	Crop swab	Cloacal swab
P	0.019^1^	0.154^b2^
Ph	0.000	0.136^b^
K	0.000	0.000^a^

^1^Crop swabs for *M. avium* were not analyzed because of too few positive samples in the experiment (for the two groups, K and Ph, *M. avium* was not detected at all, and for C it was detected in only a few samples).

^2^The different letters in the column represent a different mean share of the positive samples for birds within the two corresponding groups.
